# Selection of Optimal Nanofiltration/Reverse Osmosis (NF/RO) Membranes for the Removal of Organic Micropollutants from Drinking Water

**DOI:** 10.3390/membranes15060183

**Published:** 2025-06-17

**Authors:** E. Busra Tasdemir, Marie Pardon, Sareh Rezaei Hosseinabadi, Laurens A. J. Rutgeerts, Deirdre Cabooter, Ivo F. J. Vankelecom

**Affiliations:** 1 Membrane Technology Group (MTG), Faculty of Bioscience Engineering, KU Leuven, Celestijnenlaan 200F, P.O. Box 2454, 3001 Leuven, Belgium; 2 Laboratory of Pharmaceutical Analysis, Department of Pharmaceutical and Pharmacological Sciences, KU Leuven, Herestraat 49, P.O. Box 824, 3000 Leuven, Belgium

**Keywords:** organic micropollutants (OMPs) removal, RO and NF commercial membranes, water treatment, ultra-high-performance liquid chromatography-mass spectrometry (UHPLC-MS)

## Abstract

The growing presence of organic micropollutants (OMPs) in water sources is a major health concern. Successful removal of OMPs from water sources and ensuring the cleanliness of drinking water has become an important topic in recent years. In this study, 15 nanofiltration (NF) and reverse osmosis (RO) commercial membranes were selected and their potential to remove 10 frequently encountered OMPs in drinking water, with systematically different chemical characteristics, was evaluated. To quickly identify the most promising membranes, high throughput dead-end filtrations were initially conducted. Subsequently, the 4 best performing membranes were used in a more relevant high-throughput cross-flow filtration. Membrane performance was evaluated by analyzing OMP concentrations in the feed and retentates of the different membranes using ultra-high-performance liquid chromatography-mass spectrometry (UHPLC-MS). This study identified NF 90 (Dow), NF 270 (Dow), NFX (Synder) and TS80 (Trisep) as membranes with superior performance, with a permeance between 3 and 7 L.$m^{-2}$.h^−1^.bar^−1^ and retentions that were generally around 90%, except for NFX which showed slightly lower retentions.

## 1. Introduction

An adequate supply of freshwater has become a major global issue as a result of rising pollution and demand [1]. Surface waters are polluted by chemical sources originating from agriculture, industry and households [1,2]. Water pollution is increasingly driven by the presence of organic micropollutants (OMP) in the water resources. These pollutants, which include pharmaceuticals, pesticides and industrial chemicals (e.g., perfluorinated compounds), are collectively referred to as “contaminants of emerging concern” due to their growing prevalence in surface waters and the challenges they pose for effective drinking water purification [3,4]. To ensure clean and healthy water for human consumption, the European Council recently updated [5] the urban waste water directive on 1 March 2024 [6], and the industrial emissions directive on 12 April 2024 [7]. In these directives, the European Commission provides a watch list of chemicals to address the increasing concerns about water quality, enabling a dynamic and flexible approach to monitor priority substances [8]. It emphasizes the urgent need for efficient methods to remove OMPs from water [9]. OMPs appear in trace levels, ranging from μg.L^−1^ to ng.L^−1^ [3,10,11], and thus, require very high-performing purification techniques. Current waste water treatment plants (WWTPs) rely on conventional methods, which include primary treatment (physical removal of solids), secondary treatment (biological degradation of organic matter) and tertiary treatment (further polishing) [12]. These conventional methods lack the advanced technology to effectively eliminate OMPs to sufficiently low levels [3,9]. In other words, conventional methods are not designed to remove these compounds [11,13]. More advanced water treatment methods include granular activated carbon (GAC) adsorption, various membrane technologies and advanced chemical oxidation processes [14]. Membrane based processes [15], such as nanofiltration (NF) and reverse osmosis (RO), offer a highly promising approach for sustainable water treatment [16,17]. Research indicates that NF and RO membranes are highly effective in removing OMPs. NF membranes demonstrated significant efficacy in removing polyfluoroalkyl substances (PFAS) from water [18,19,20,21,22]. In one of the studies, the rejection of thirteen OMPs, including acetaminophen, carbamazepine, ibuprofen and diclofenac, by four commercial flat sheet membranes, i.e., NF1 (<200 Da), NF2 (268 Da), NF6 (516 Da), RO4 (<200 Da) and NF270 (290 Da), was evaluated. Ionic OMPs were strongly affected by electrostatic repulsion from the membrane surface, while non-ionic OMPs were more affected by size exclusion and hydrophobic–hydrophilic interactions [23]. Another set of commercial NF membranes for removal of pollutants included NFX, NFG, NF270, NF90, DK, DL, TS80, SB90, TS40, XN45, NPO30 and NPO10 (molecular weight cut-off [MWCO] ranges of 150 to 1200 Da) [24]. Higher pressure increased water production efficiency but led to more fouling, particularly in NF90. Increased pH caused an increase in efficiency for charged pollutant removal while decreasing the removal of ammonium. Among all tested membranes, NF90 and TS80 membranes exhibited the most promising performance, with NF90 being especially successful in removing heavy metals and PFAS [24]. The impact of fouling on the retention of sulfamethoxazole, ibuprofen and carbamazepine was studied using NF membranes NF90, NF270 and TFC-SR2. NF90 achieved ~98% retention for carbamazepine, while NF270 reached 80% retention [25]. The rejection of organic molecules was further investigated using an ultra-low-pressure RO membrane (ES20). Rejection could be correlated with pKa values [25] while hydrophobicity was identified as a critical parameter influencing the retention of OMPs. Hydrophobic compounds tended to permeate more easily, while those with high affinity for the aqueous phase (thus, low log K_ow_ values) were more likely to be retained [26,27]. Osorio et al. studied 58 OMPs and grouped them based on their molecular characteristics [18], which was crucial in enhancing the development of models to more accurately predict OMP removal by membranes. These studies, using a variety of conditions, confirmed that OMP rejection is based on a set of complex interactions depending on the intrinsic properties of solutes and membranes, solution chemistry and operating conditions.

In the current study, 15 commercial membranes with a MWCO in the RO/NF range were selected based on their promising performance in water purification [28]. These membranes were screened for their potential to reject specific OMPs, which were chosen to encompass all OMP types described by Osorio et al. [18]. The compounds were categorized by their MW and chemical structure, taking into account a combination of hydrophobic, hydrophilic and ionic characteristics. The selected OMPs were then mixed in tap water to prepare a unified feed solution for analysis. An advanced UHPLC-MS method was developed to precisely determine the concentration of each OMP in the feed and retentate of the different membranes. This technique provides accurate measurements for all compounds of interest, including their concentration and potential compositional variations, both before and after membrane filtration. The objective of this work was thus, to show the potential of the 15 tested commercially available membranes to remove a range of representative OMPs, which broadly covered a range of relevant molecular characteristics (i.e., Log K_ow_, pKa, logD, MW) and to select the most appropriate membranes.

## 2. Materials and Methods

### 2.1. OMP Selection

The selected OMPs and their physico-chemical properties are shown in Table 1. OMPs were selected based on their frequent detection in wastewater. A more detailed classification, as described by Osorio et al. [18], was used to better analyze the rejection mechanism of OMPs.

For the current study, OMPs were categorized into 8 groups based on their physicochemical properties. While each group reflects a dominant characteristic, some compounds exhibit overlapping properties and can therefore fit into more than one group. The following compounds were selected to represent one group each: Group 1 (Iodinated compounds): Iopromide, Group 2 (Perfluorinated compounds): Perfluorooctanoic acid (PFOA), Group 3 (Cyclic, hydrophobic compounds): Ibuprofen, Group 4 (Cyclic, charged compounds): Diclofenac and Sertraline, Group 5 (Nitrogen rich compounds): Metformin, Caffeine, Group 6 (Moderate MW, hydrophobic compounds): Carbamazepine, Group 7 (Small, neutral compounds): Acetaminophen (Paracetamol), Group 8 (Small, hydrophilic compounds): Gabapentine. The main purpose of this grouping is to select a specific set of probe molecules, which represent all types of OMPs, thus enabling general conclusions to be made about the overall performance of the screened membranes towards all possible OMPs.

PFOA and caffeine were from Sigma-Aldrich (Diegem, Belgium), ibuprofen was from Certa (Braine-l’Alleud, Belgium), diclofenac, carbamazepine and paracetamol were from Alpha Pharma (Zwevegem, Belgium), gabapentine and metformin were from Fluka (Seelze, Germany) and iopromide and sertraline were available in the lab as USP reference standards. For the LC-MS analysis of the OMPs (see Section 2.4), the following internal standards were used: ^2^H6-metformin and ^2^H3-iopromide from LGC (Teddington, UK), ^13^C3-gabapentine, ^13^C3-caffeine, ^2^H3-ibuprofen, ^2^H10-carbamazepine, ^2^H4-paracetamol and ^13^C6-diclofenac from Sigma-Aldrich, ^13^C6-sertraline from Alsachim (Illkirch Graffenstaden, France) and ^13^C8-PFOA from Agilent Technologies (Waldbronn, Germany). All molecular descriptors shown in Table 1 (log K_ow_, log D, pKa and MW) were calculated using the Chemicalize Software (ChemAxon).

### 2.2. Selection of Commercial NF and RO Membranes

Nine typical NF membranes and six typical commercial RO membranes were selected to be used for OMP removal. Table 2 presents the MWCO, contact angle, water permeability, roughness and zeta potential of the membranes, as found in the literature. The surface charge of the membranes is described as the zeta potential and contact angles represent the hydrophobicity/hydrophilicity of the membrane surface [29].

### 2.3. Filtration Experiments

**Figure 1:**
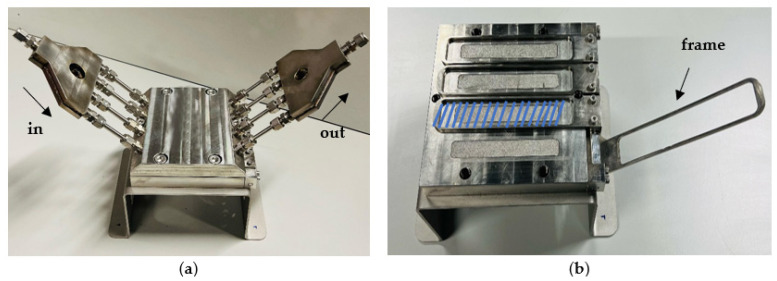
High throughput cross-flow module: (**a**) the module in its closed state, and (**b**) the membrane placement area, indicated by the blue zone.

Filtrations were conducted using two different filtration systems. Initially, a high-throughput dead-end system [49] was used, enabling rapid simultaneous filtration of up to 16 coupons, each with an active area of $1.54\;\times 10^{-4}$ m^2^ per coupon under a pressure of 10 bar at room temperature (RT). Initially, a 3 h filtration using tap water, sourced from the University Hospital of Leuven (UZ Leuven, Leuven, Belgium), was performed to allow the membranes to reach steady-state. Hereafter, permeates were collected from the 15 different commercial membranes. Next, the feed tank was filled with a solution containing a mixture of the 10 OMPs, which was thoroughly stirred. The feed solution was prepared by diluting stock solutions of the OMPs into the hospital tap water to achieve the desired concentrations which will be shown in Section 3.1. The system was operated for 3 more hours, after which permeates were collected again. The second set of filtrations was performed in a high throughput cross-flow filtration setup [50]. For this experiment, only the best performing membranes from the first experiment were selected. As illustrated in Figure 1, the system consisted of 4 separate parallel cells, mounted in a module, each equipped with a positioning frame to secure the membrane [50]. The experiments were carried out under similar conditions (including feed concentration) to the dead-end filtration. The active membrane area for each membrane was $1.6\;\times \;10^{-3}\;m^{2}$.

Equation (1) was used to calculate the permeance *P*$\;\left(L.m^{-2}.h^{-1}.{\mathrm{bar}}^{-1}\right)$, where *V (L)* is the permeate volume, *A* (m^2^) the membrane area, *t* (h) the filtration time and Δ*P* (bar) the applied pressure:

(1) $$P=\frac{V}{A\times t\times \;\Delta P\;}$$

Using Equation (2) the retention was calculated, where $C_{f}$ and $C_{p}$ represent the solute concentration (µg/L) in the feed and permeate, respectively, as determined by LC-MS (see Section 2.4).

(2) $$R=\frac{C_{f}-C_{p}}{C_{f}}\times 100$$

### 2.4. Permeate Analysis

#### 2.4.1. Instrumentation

LC-MS analyses of the feed and retentate samples were carried out on a 1290 Agilent Infinity II 2D-LC system from Agilent Technologies, equipped with two 1290 high-pressure binary pumps, a 1290 autosampler with a maximum injection volume of 20 µL, a Multicolumn Thermostat (MCT) column oven and two diode array detectors (DAD) with a 1.0 µL flow cell and a path length of 10 mm. Only the first dimension of the LC system (without DAD) was used for the analysis. The LC system was coupled to a 6530 Q-TOF MS with a jet stream electrospray ionisation source (Agilent Technologies, Waldbronn, Germany). Agilent OpenLab CDS Chemstation edition (Rev. C.01.10 [287]) software and Agilent 1290 Infinity 2D-LC software add-on (version A.01.04 [036]) were used to control the LC system. Agilent MassHunter Workstation Data Acquisition software was used to operate the MS system. Data were analyzed using Agilent 1290 Infinity 2D-LC software, the MassHunter Qualitative Analysis 10.0 software and Microsoft Excel.

Electrical conductivity and pH were measured using a Consort multi parameter analyzer C3010 SK10T and VWR pHenomenal pH meter (a pH electrode combined with an integrated temperature probe, PEEK shaft, fixed cable and ISM), respectively.

#### 2.4.2. Analytical Methods

The solvents used to prepare the mobile phases for the LC-MS analyses were acetonitrile (ACN, MS grade) from Biosolve (Dieuze, France), and water (H_2_O, MS grade) from Thermo Fisher Scientific. Ammonium fluoride (AF, ≥99.99%) was from Sigma-Aldrich (Steinheim, Germany), and formic acid (FA, 99% for LC-MS) was from VWR (Leuven, Belgium). LC-MS separations were performed using the following mobile phases: (A) 0.1% FA in H_2_O, (B) 0.1% FA in ACN, (C) 2 mM AF in H_2_O and (D) ACN. All mobile phases were degassed with helium prior to use.

A Zorbax Stable-bond C_18_ column (2.1 × 100 mm; d_p_ = 1.8 µm) from Agilent Technologies was used for separations in both positive and negative MS ionization mode. In positive ionization mode, the mobile phase was varied as follows: 99/1-1/99-99/1-99/1 (*v*/*v*%) A/B in 0-6-6.6-8 min at a flow rate of 0.4 mL/min. In negative ionization mode, mobile phases C and D were used with the same gradient program and flow rate. The column was operated at 30 °C, and the injection volume was set to 2 µL. The following ionisation source conditions were applied: drying gas flow rate 12 L/h, drying gas temperature 300 °C, sheath gas flow rate 11 L/h, sheath gas temperature 350 °C, capillary voltage 3.5 (+) and 2.5 (-) kV, nebulizer gas pressure 25 (+) and 45 (-) psi and nozzle voltage 1.5 kV. The MS was operated in 2 GHz Extended Dynamic Range mode. MS spectra were collected at 3 spectra/s. Purine (*m*/*z* 121.0508) and hexakis (*m*/*z* 922.0097) in positive ionization mode, and TFA anion (*m*/*z* 112.9855) and hexakis (*m*/*z* 1033.9881) in negative ionization mode, were used as the external lock mass for real-time mass accuracy correction. Each analysis was performed in triplicate.

Internal standards (^2^H- or ^13^C-) were added as a mixture to the samples before LC-MS analysis (10 µL of the internal standard mixture was added to 90 µL of each sample), and used for accurate quantification. The final concentration of the internal standards in the resulting sample was set at an intermediate concentration of the calibration curve for each compound. The developed method was evaluated for linearity at 5 different concentrations between the lower limit of quantification (LLOQ) and the upper limit of quantification (ULOQ), as shown in Table 3. Repeatability was assessed for 3 repeated injections at an intermediate concentration of the calibration range for each compound. The limit of detection (LOD) and LLOQ were determined as the concentrations for which the S/N was ≥3 or 10, respectively.

As can be deduced from Table 3, linearity (R^2^ > 0.9897) was observed for all compounds in their evaluated range, while RSD values for 3 repeated injections were always below 4%. LOD and LLOQ values are also shown in Table 3, and are generally in the µg/L-range. Note that the concentrations in the feed solutions used for the filtration experiments (see Section 3.1) were, for each compound, 100× higher than the LLOQ-values to allow measuring a retention percentage of at least 99%. These concentrations are somewhat higher than relevant environmental concentrations, but nonetheless allow an intrinsic comparison of the evaluated membranes.

## 3. Results and Discussion

### 3.1. OMP Retention Mechanism

OMPs were classified based on their molecular properties, including MW, octanol-water partition coefficient (Log K_ow_), log D and pKa, to better understand the removal mechanisms influencing rejection efficiency in membrane-based processes. One of the key principles in membrane separation is the retention of organic compounds having a molar mass exceeding the membrane MWCO [51]. This well-defined mechanism is often, however, an over-simplification for RO/NF and can be influenced or even completely over-ruled by additional factors, such as electrostatic repulsion or specific solute–membrane interactions [18]. Hydrophobicity is often defined by log K_ow_ or log D. Whereas log K_ow_ is only used for molecules in their neutral form, all ionized and non-ionized species are expressed by their log D. For non-ionized species, the log K_ow_ value is equal to the log D value [19]. NF and RO membranes are considered to be less hydrophilic than water, therefore, the solubility of hydrophobic solutes (with a large value of log K_ow_ or log D) in the membrane will be enhanced, which should decrease retention [18]. Dissociation and charge carrying capacity of molecules along with hydrophobicity are known to significantly affect OMP retention [18].

Initially, a high-throughput dead-end filtration setup was for fast screening. The purpose of this was to demonstrate the filtration behavior of the membranes under the applied conditions, to determine the membranes that exhibited the best performance and to provide a baseline performance before performing more relevant but also more time-, labor- and resource-intensive cross-flow filtrations.

**Figure 2:**
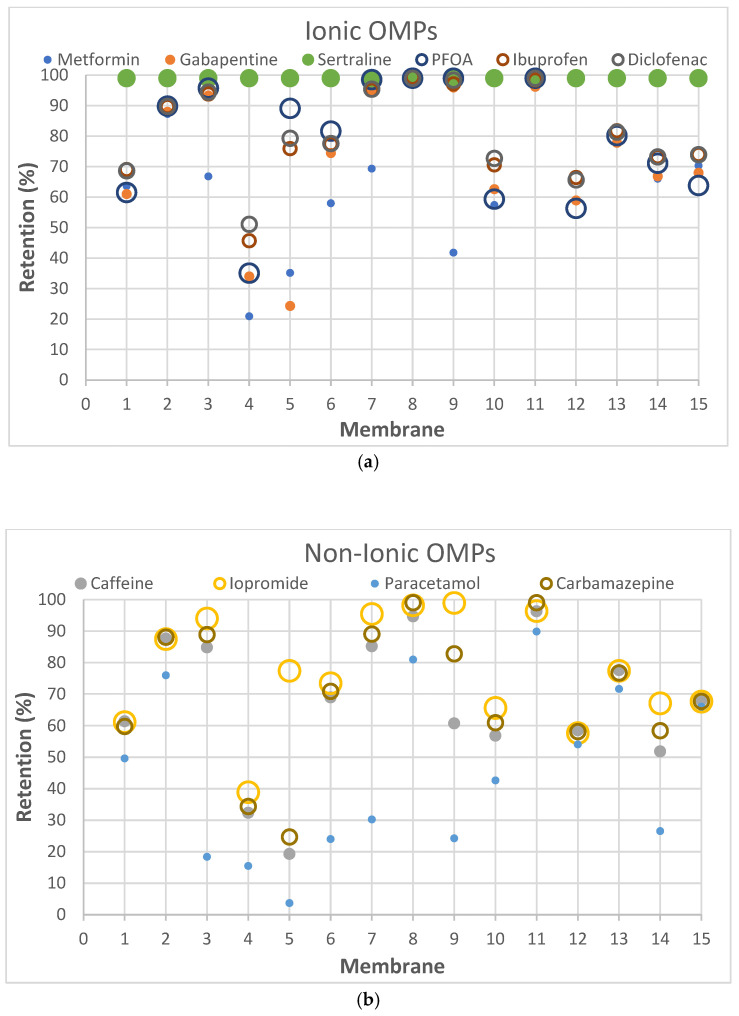
Relationship per membrane type between MW (reflected in the size of the dots) and retention of (**a**) ionic OMPs and (**b**) non-ionic OMPs. The X-axis shows, respectively; 1-NanoPro A-3012 (Unisol), 2-NF90 (Dow), 3-NF270 (Dow), 4-Duracid NF (GE Osmonics), 5-HYDRACoRe 7470 pHT (Nitto Hydranautics), 6-NFX Membrane (Synder), 7-TS80 (Trisep), 8-SB90 (Trisep), 9-TS40 (Trisep), 10-Filmtec (DOW), 11-Filmtec BW30 (DOW), 12-ESPA4 (Nitto Hydranautics), 13-X201 (Trisep), 14-SB50 (Trisep), 15-UTC-73AC (Toray).

According to the results obtained in dead-end filtration (Table 4), iopromide (791 g.mol^−1^) and PFOA (414 g.mol^−1^) did not exhibit the expected high retentions for all selected membranes, despite their high MW, confirming the fact that the MWCO-value for a membrane is not the primary determining parameter in this case. The sole use of the MWCO can thus be misleading as some OMPs exhibit lower rejection than expected based on MW alone [52,53]. This is also clearly illustrated as a function of MW per membrane type for ionic and non-ionic OMPs in Figure 2 where the largest dots do not always correspond to the highest rejections.

Very striking was that sertraline exhibited >97% retention with all tested membranes, suggesting that sertraline was retained to a great extent, independent of the type of membrane used. It is one of the OMPs with the highest MW in this study, but is especially also a rigid and bulky molecule. As its log K_ow_ (5.11) and log D (2.67) indicate, sertraline is also among the most hydrophobic compounds in this study. This high hydrophobicity could also help explain the high sertraline retention values by this set of relatively polar membranes. However, it must be noted that some degree of degradation over time was observed for sertraline, which could also explain the observed values, which relied on measured concentrations in the permeate of the non-degraded compound. Likewise, PFOA exhibited ≥99% retention in membranes 8, 9 and 11.

Remarkably, membrane 11 (Filmtec BW30) achieved ≥99% retention, not only for PFOA and sertraline, but also for diclofenac and carbamazepine. This indicates that membrane 11 has an overall higher separation efficiency compared to the other membranes. RO membranes, due to their denser selective layer, indeed typically exhibit a higher selectivity than NF membranes [49,50]. However, the high selectivity for RO-membranes in comparison with NF-membranes is not really visible for the selected set of OMPs in Table 4 or Figure 2.

The fact that paracetamol, which belongs to group 7 (small, neutral compounds), exhibits a low retention for all membranes, suggests that paracetamol may be carried with the permeating water through the membrane, largely without really interacting with the membrane material as such. Its low log K_ow_ (0.91) and log D (0.91) values confirm this very low hydrophobicity.

Electrostatic repulsion becomes especially significant when OMP molecular sizes are comparable to or smaller than the membrane’s MWCO. As mentioned, MWCO is not always a reliable parameter for making comparisons because it does not take into account factors such as solute solvation, solute shape and charge [54]. The interactions between membrane and OMP charges can be crucial when MWCOs are similar. The membrane zeta potential, influenced by pH and ionic strength [52], reflects this potential charge interaction. All membranes in this study exhibited negative zeta potentials at pH 7 (Table 2). Understanding OMP pKa values, in addition to membrane charges, helps elucidate rejection mechanisms. Oppositely charged OMPs may exhibit increased concentration at the membrane surface due to attraction, while similarly charged OMPs experience repulsion. Ibuprofen (average retention ~80%), diclofenac (~81%) and PFOA (~79%) are all negatively charged and demonstrated high retention. Conversely, metformin (~65%), positively charged, showed lower retention. Metformin’s lower MW (129 g.mol^−1^) compared to sertraline (306 g.mol^−1^) also contributed to its lower rejection.

Ionic OMPs generally showed higher retentions than non-ionic OMPs, as visualized by the overall higher positions of dots in Figure 2a versus Figure 2b. If the molecular weight is similar, the electrical charge of the molecules significantly affects the retention rate: negatively charged compounds generally have higher rejection rates and positively charged compounds tend to have slightly lower retention [55], in line with the mostly negative charges on the membrane. This is supported by the observation that negatively charged ibuprofen and diclofenac, which have similar molecular weights, show higher retention compared to positively charged metformin.

Membrane hydrophobicity, indicated by the water contact angle, also influences OMP retention. SB90, with a high contact angle (59°), TS40 with a highly negative zeta potential (–52 mV) and BW30 with a high contact angle (90.3°) and slightly negative zeta potential (–10 mV at pH 9), all demonstrated a high retention for OMPs with high log K_ow_ values: PFOA (Mw 414 g.mol^−1^ and log D 1.58), ibuprofen (Mw 206 g.mol^−1^ and log D 1.71), diclofenac (Mw 296 g.mol^−1^ and log D 1.37) and sertraline (Mw 306 g.mol^−1^ and log D 2.67). However, some studies suggest that high hydrophobicity can lower retention, especially for OMPs with sizes comparable to the membrane’s MWCO [26].

### 3.2. Membrane Selection and Overall Performance Evaluation

To identify the best-performing membranes for OMP removal from drinking water, overall OMP retentions and water permeances were evaluated.

**Figure 3:**
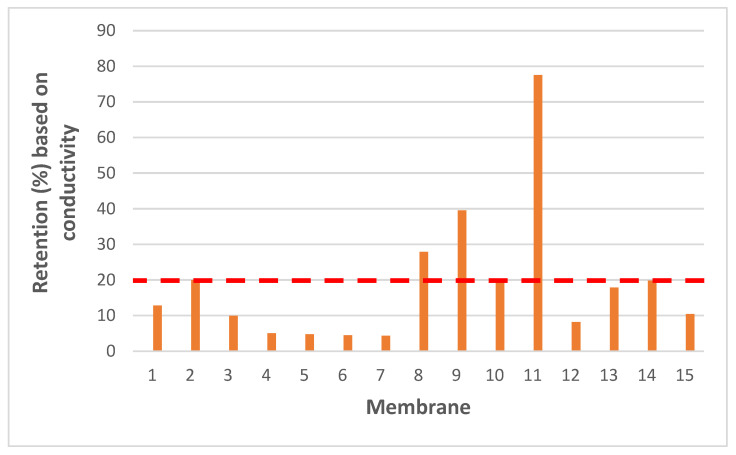
Retention (%) based on conductivity for the tested membranes [Tap water conductivity was measured as 756 µS.cm^–1^]. X-axis shows, respectively; 1-NanoPro A-3012 (Unisol), 2-NF90 (Dow), 3-NF270 (Dow), 4-Duracid NF (GE Osmonics), 5-HYDRACoRe 7470 pHT (Nitto Hydranautics), 6-NFX Membrane (Synder), 7-TS80 (Trisep), 8-SB90 (Trisep), 9-TS40 (Trisep), 10-Filmtec (DOW), 11-Filmtec BW30 (DOW), 12-ESPA4 (Nitto Hydranautics), 13-X201 (Trisep), 14-SB50 (Trisep), 15-UTC-73AC (Toray).

Ideally, only OMPs should be removed, while salts should not be retained, as they would otherwise need to be added again in a further step to make the water potable. Therefore, conductivity-based retentions were also determined for each membrane. Low values are thus, desired here to reflect sufficient passage of salts that are present in drinking water. As seen in Figure 3, several membranes show rather high retentions, e.g., BW30 exhibited the highest retention based on conductivity measurements, which is consistent for a membrane with RO characteristics. For this reason, only membranes with conductivity-based retention values below 20% were selected as a practical threshold to ensure minimal salt rejections for further experiments.

**Figure 4:**
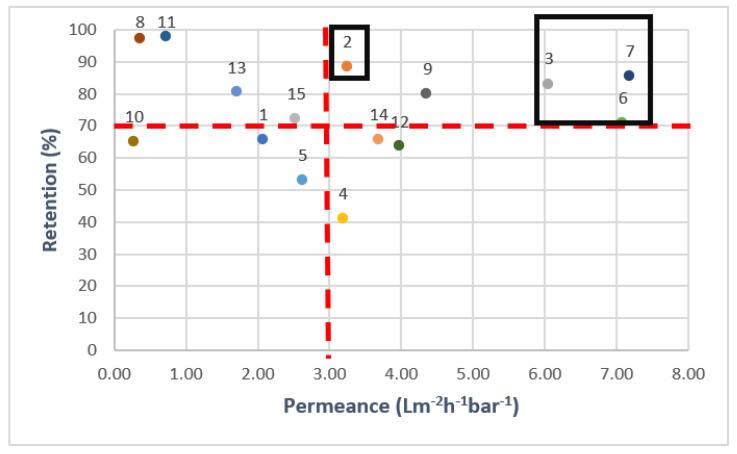
Average OMP retentions and permeances for all commercial membranes. Black boxes are drawn to show the selected membranes for cross-flow filtration. 1-NanoPro A-3012 (Unisol), 2-NF90 (Dow), 3-NF270 (Dow), 4-Duracid NF (GE Osmonics), 5-HYDRACoRe 7470 pHT (Nitto Hydranautics), 6-NFX Membrane (Synder), 7-TS80 (Trisep), 8-SB90 (Trisep), 9-TS40 (Trisep), 10-Filmtec (DOW), 11-Filmtec BW30 (DOW), 12-ESPA4 (Nitto Hydranautics), 13-X201 (Trisep), 14-SB50 (Trisep), 15-UTC-73AC (Toray).

Another important consideration is the balance between OMP retention and permeance. Therefore, the results of the filtration experiments were plotted in a Robeson-like plot [56], which can be seen in Figure 4. To make the data analysis clearer, the mathematical average of all separate OMP retentions was used to indicate the overall performance of the membranes. As the order of the retentions of the individuals OMPs was mainly the same for each membrane, the use of this combined retention parameter was considered acceptable to create a good overall understanding of the membrane performance. Only the membranes with an average retention above 70% (according to the OMP feed solution concentrations) and high permeance (above 3 L.m^−2^.h^−1^.bar^−1^, in line with data presented in Table 2) were thus selected for the next experiments. Membrane 9 was not included in this selection due to its high retention based on conductivity.

**Figure 5:**
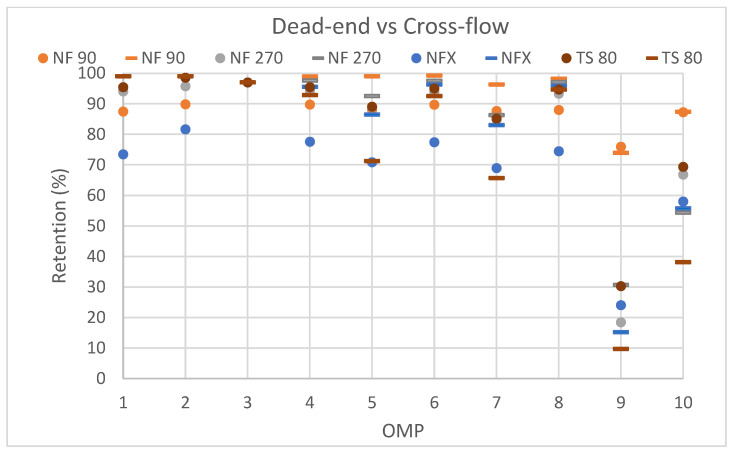
Comparison of dead-end filtration and cross-flow filtration for the selected membranes: 2 (NF 90), 3 (NF270), 6 (NFX) and 7 (TS 80). Dead-end results are shown with dots, while cross-flow results are indicated with a dash. X-axis shows, respectively; 1-Iopromide, 2-PFOA, 3- Sertraline, 4-Diclofenac, 5-Carbamazepine, 6-Ibuprofen, 7-Caffeine, 8-Gabapentine, 9-Acetaminophen (Paracetamol), 10-Metformin.

Next, a cross-flow filtration was performed with the selected membranes (NF90, NF270, NFX and TS80) with the results shown in Figure 5. In general, it can be seen that both dead-end and cross-flow filtration modes yielded comparable results. The fact that retentions remained largely the same for cross-flow compared to dead-end filtration indicates that the latter did not suffer from concentration polarization, which is not unexpected for the rather low solute concentrations (in Table 4) that were used here and that are characteristic of OMPs.

## 4. Conclusions

Commercial RO and NF membranes were used successfully to remove OMPs within acceptable ranges while not retaining salts excessively. Based on the OMP retentions and water fluxes, NF90 (Dow), NF270 (Dow), NFX (Synder) and TS80 (Trisep) were determined as the most promising membranes. The highest permeance values were obtained in the following order: TS80 (Trisep) > NFX (Synder) > NF270 (Dow) > NF90 (Dow), while the highest retention values were found in the order of NF90 (Dow) > TS80 (Trisep) > NF270 (Dow) > NFX (Synder), respectively. All 4 are classified as NF membranes and have a polyamide selective layer. For the studied conditions, dead-end filtrations showed a similar performance as cross-flow. Some OMPs, such as paracetamol, were more difficult to remove than others, the concentrations of which can be decreased drastically in one single NF process.

## Figures and Tables

**Table 1:** List of selected OMPs which were categorized into 8 groups based on their physico-chemical properties (i.e., MW, octanol-water partition coefficient (Log K_ow_), log D and pKa).

Group	Compound	log K_ow_	log D *	pKa	Molecular Formula	MW (g/mol)	Structure
1	Iopromide	−0.4	−0.44	11.1	C_18_H_24_I_3_N_3_O_8_	791	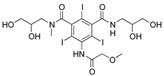
2	PFOA	5.1	1.58	0.3	C_8_HF_15_O_2_	414	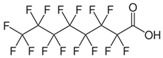
4	Sertraline	5.1	2.67	-	C_17_H_17_Cl_2_N	306	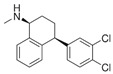
4	Diclofenac	4.2	1.37	4.0	C_14_H_11_Cl_2_NO_2_	296	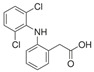
6	Carbamazepine	2.7	2.77	15.9	C_15_H_12_N_2_O	236	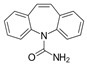
3	Ibuprofen	3.8	1.71	4.8	C_13_H_18_O_2_	206	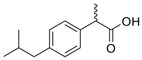
5	Caffeine	−0.5	−0.55	-	C_8_H_10_N_4_O_2_	194	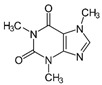
8	Gabapentine	0.9	−1.27	4.6	C_9_H_17_NO_2_	171	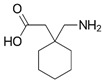
7	Acetaminophen (Paracetamol)	0.9	0.91	9.4	C_8_H_9_NO_2_	151	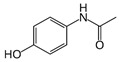
5	Metformin	−0.9	−5.69	-	C_4_H_11_N_5_	129	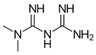

* pH = 7.

**Table 2:** List of selected physicochemical and structural properties (i.e., type, selective layer, MWCO, contact angle, water permeability, roughness and zeta potential) of the membranes used in this study.

No	Membrane	Membrane Type	Selective Layer **	MWCO (Da)	Contact Angle (°)	Water Permeability $(L.m^{-2}.h^{-1}.bar^{-1}$)	Roughness (nm)	Zeta Potential (mV) *	Reference
1	NanoPro A-3012 (Unisol)	NF	Composite material	200	-	2.12	58	−14	[30,31]
2	NF90 (Dow)	NF	Polyamide-TFC	200	58	8.67	63.9	−48	[32,33,34]
3	NF270 (Dow)	NF	Polyamide	170	27	8.5	28 µm	−19	[35]
4	Duracid NF (GE Osmonics)	NF	Polyamide-TFC	146	35	1.8	7.7	−80	[36]
5	HYDRACoRe 7470 pHT (Nitto Hydranautics)	NF	Sulfonated polyethersulfone	200–250	62	4.3	-	-	[37,38,39]
6	NFX Membrane (Synder)	NF	Polyamide-TFC	150–300	17	4.21	-	−25	[34]
7	TS80 (Trisep)	NF	Polyamide-TFC	150	29.8	6.5	79.4	−32 (at pH = 9)	[40,41]
8	SB90 (Trisep)	NF	Cellulose Acetate	150	59	2.8	9.8	-	[24,38,42]
9	TS40 (Trisep)	NF	Polypiperazine-amide	200–300	30	6.1	11	−52	[43]
10	Filmtec (DOW)	RO	Polypiperazine-amide TFC	200	55	-	-	−30	[39]
11	Filmtec BW30 (DOW)	RO	Polyamide	~100	90.3	5	68.3	−10 (at pH = 9)	[41,44]
12	ESPA4 (Nitto Hydranautics)	RO	Composite Polyamide	-	-	6.48	-	-	[45]
13	X201 (Trisep)	RO	Polyamide-urea-TFC	-	106.8	3.33	69.5	-	[46]
14	SB50 (Trisep)	RO	Cellulose Acetate	152	-	2.18	-	−13	[47]
15	UTC-73AC (Toray)	RO	Polyamide-TFC	-	40.3	3.4	37.3	-	[48]

* pH ~ 7; ** With all details made available on the suppliers’ websites.

**Table 3:** Validation parameters for the developed LC-MS method, showing linearity, repeatability (as the % RSD of 3 repeated injections at an intermediate concentration of the calibration curve), LOD and LLOQ.

Compound	Linear Range (µg/L)	R^2^	RSD (%)	LOD (µg/L)	LLOQ (µg/L)
Iopromide	31.3–25000	0.9995	2.4	7.8	31.3
PFOA	0.6–500	0.9981	2.5	0.2	0.6
Ibuprofen	7.8–6250	1.0000	1.6	2.0	7.8
Diclofenac	8.1–6250	0.9998	3.7	2.0	7.8
Sertraline	1.6–1250	0.9897	1.6	0.8	1.6
Gabapentine	6.3–5000	0.9998	1.4	1.6	6.3
Metformin	0.8–625	0.9999	2.8	0.4	0.8
Carbamazepine	12.5–10000	0.9986	1.1	6.3	12.5
Caffeine	50–40000	1.000	0.5	12.5	50
Paracetamol	12.5–10000	0.9997	3.7	3.1	12.5

**Table 4:** Mw (g/mol), feed solution (µg/L), conductivity (µs/cm) and retention (%) of OMPs by selected RO and NF membranes in dead-end filtrations 1- NanoPro A-3012 (Unisol), 2-NF90 (Dow), 3-NF270 (Dow), 4-Duracid NF (GE Osmonics), 5-HYDRACoRe 7470 pHT (Nitto Hydranautics), 6-NFX Membrane (Synder), 7-TS80 (Trisep), 8-SB90 (Trisep), 9-TS40 (Trisep), 10-Filmtec (DOW), 11-Filmtec BW30 (DOW), 12-ESPA4 (Nitto Hydranautics), 13-X201 (Trisep), 14-SB50 (Trisep), 15-UTC-73AC (Toray).

			Retention %														
			NF									RO					
OMP	Mw (g mol^−1^)	Feed (µg/L)	1	2	3	4	5	6	7	8	9	10	11	12	13	14	15
Iopromide	791	3120	61	87	94	39	77	73	95	98	99	66	96	58	77	67	68
PFOA	414	60	62	90	96	35	89	82	99	≥99	≥99	59	≥99	56	80	71	64
Sertraline	306	150	≥97	≥97	≥97	≥97	≥97	≥97	≥97	≥97	≥97	≥97	≥97	≥97	≥97	≥97	≥97
Diclofenac	296	780	69	90	94	51	79	78	95	≥99	98	73	≥99	66	81	73	74
Carbamazepine	236	2500	60	88	89	34	25	71	89	≥99	83	61	≥99	58	77	58	68
Ibuprofen	206	780	69	90	94	46	76	77	95	99	97	71	98	66	82	73	74
Caffeine	194	5000	61	88	85	32	19	69	85	95	61	57	96	59	78	52	68
Gabapentine	171	620	61	88	93	34	24	74	95	98	96	63	96	59	78	67	68
Paracetamol	151	1250	50	76	18	15	4	24	30	81	24	43	90	54	72	27	66
Metformin	129	30	64	87	67	21	35	58	69	98	42	57	98	59	78	66	70
Water permeance (LMH/bar)			2.08	3.25	6.06	3.19	2.63	7.09	7.19	0.36	4.37	0.28	0.73	3.98	1.71	3.69	2.54
Conductivity (µs/cm)		756	659	605	681	718	720	722	723	545	457	605	170	694	621	606	677
MWCO (Da)			200	200	170	146	200–250	150–300	150	150	200–300	200	~100	-	-	152	-

## Data Availability

The original contributions presented in this study are included in the article/Supplementary Material. Further inquiries can be directed to the corresponding author(s).

## Supplementary Materials

The following supporting information can be downloaded at https://www.mdpi.com/article/10.3390/membranes15060183/s1, Figure S1: positive mode MS, B: negative mode MS.
