# Supplementary material for: Selection of Optimal Nanofiltration/Reverse Osmosis (NF/RO) Membranes for the Removal of Organic Micropollutants from Drinking Water
Source: Membranes (Basel). 2025 Jun 17;15(6):183. doi: 10.3390/membranes15060183 (PMC12195043; doi:10.3390/membranes15060183)
Supplement: Supplementary file 1 [file membranes-15-00183-s001.zip › membranes-3682755-supplementary.pdf]

Selection of optimal Nanofiltration/Reverse Osmosis (NF/RO) membranes for the removal of organic micropollutants from drinking water

E. Busra Tasdemir[1], Marie Pardon[2], Sareh Rezaei Hosseinabadi[1], Laurens A.J. Rutgeerts[1], Deirdre Cabooter[2], Ivo F.J. Vankelecom[1]*

[1] *Membrane Technology group (MTG), Faculty of Bioscience Engineering, KU Leuven, Celestijnenlaan 200F, Box 2454, 3001 Leuven, Belgium*

[2] *Laboratory of Pharmaceutical Analysis, Department of Pharmaceutical and Pharmacological Sciences, KU Leuven, Herestraat 49, box 824, 3000 Leuven, Belgium*

* Corresponding author: ivo.vankelecom@kuleuven.be

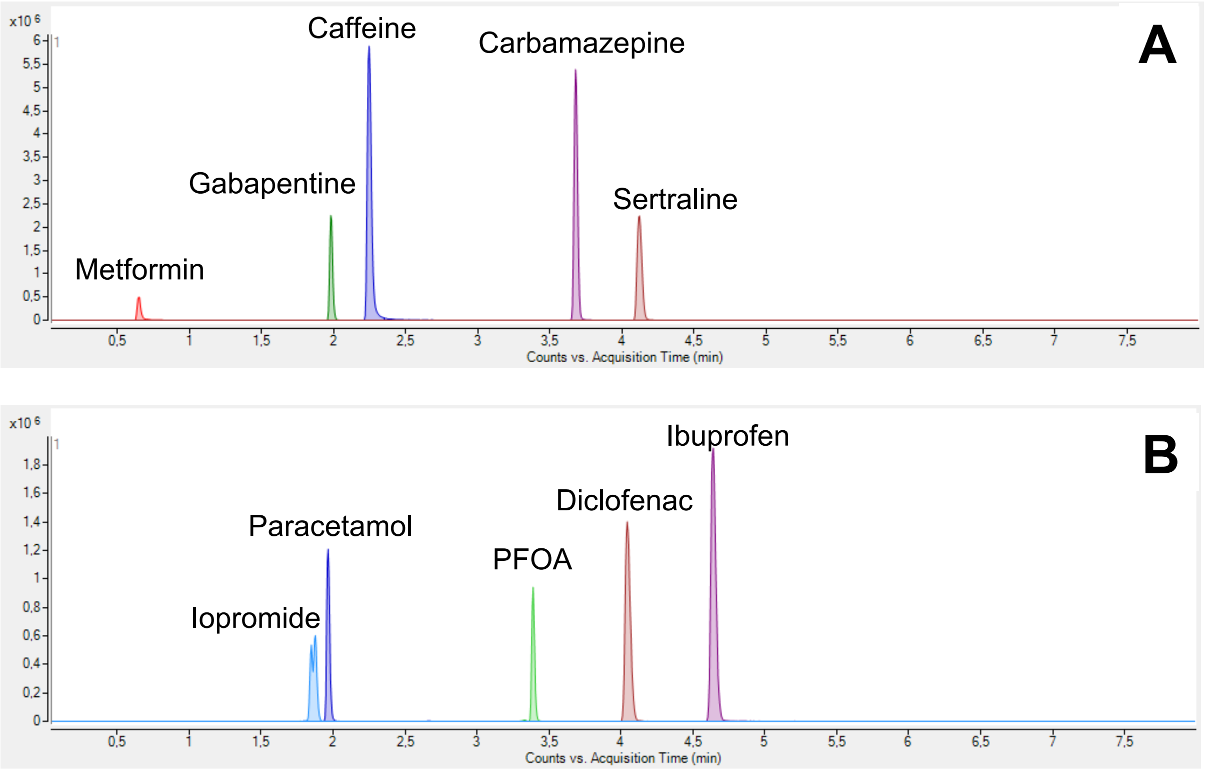

Figure S1.: positive mode MS, B: negative mode MS
